# Trehalose metabolism: an integrative hub linking energy homeostasis and development in insects

**DOI:** 10.3389/finsc.2026.1872741

**Published:** 2026-07-28

**Authors:** Sharada D. Mohite, Rakesh S. Joshi

**Affiliations:** 1 Biochemical Sciences Division, CSIR-National Chemical Laboratory, Pune, Maharashtra, India; 2 Academy of Scientific and Innovative Research (AcSIR), Ghaziabad, Uttar Pradesh, India

**Keywords:** development, homeostasis, hormones, metabolism, trehalose

## Abstract

Trehalose metabolism in insects represents an evolutionarily conserved metabolic hub regulated by endocrine pathways that couple energy balance to the successful execution and robustness of developmental transition. As the principal circulating sugar in insects, trehalose exhibits unique physicochemical properties that confer both metabolic efficiency and resilience to environmental stress, positioning it as a central currency in insect physiology. Emerging evidence reveals that trehalose metabolism operates as a dynamic interface linking endocrine signaling, nutrient sensing, and developmental transitions. Through coordinated interactions with regulatory hormonal systems such as adipokinetic hormone, insulin-like peptides, juvenile hormone, and ecdysteroids, trehalose metabolism is closely linked to nutrient-sensing pathways, including AMPK, TOR, and FoxO, that collectively shape growth, metamorphosis, and stress tolerance. Perturbations in trehalose metabolic enzymes consistently disrupt developmental homeostasis, leading to defects in molting, reproduction, and survival. Overall, these findings support the view that trehalose is a key energy substrate that plausibly integrates endocrine regulation, nutrient status, and developmental transitions. Emerging evidence suggests that trehalose metabolism may contribute to the metabolic conditions influencing life-history transitions, although further mechanistic studies are needed to establish its signaling functions. By synthesizing recent advances across molecular, physiological, and ecological scales, this review highlights trehalose metabolism as a critical nexus in insect developmental biology and a promising, yet underexploited, target for next-generation pest management strategies.

## 1 Introduction: trehalose beyond an energy sugar

Insects rely on trehalose not simply as a circulating carbohydrate, but as a metabolite that integrates physiological state with developmental and environmental demands. Unlike vertebrates, where glucose dominates systemic energy distribution, insects have evolutionarily favored trehalose as their principal hemolymph sugar (1–4). This distinction reflects more than biochemical convenience; it points to a fundamentally different strategy for maintaining metabolic stability in an open circulatory system, where tissues are continuously exposed to circulating metabolites and fluctuations in the internal milieu. Trehalose makes up a major portion of the overall sugar content in insect hemolymph, with amounts exceeding 90% in various orders, including Lepidoptera, Diptera, Orthoptera, and Hymenoptera, while its quantity may be 10-to 100-fold higher than that of glucose (4, 5). In the middle of the 20th century, trehalose was first isolated from pupal hemolymph of the silk moth *Telea polyphemus*, and classified as a transportable sugar that plays an important role in providing energy for energy-consuming activities, such as flying, locomotion, development, and reproduction (4, 6). However, recent studies have demonstrated that trehalose metabolism is a multifunctional process regulated through the integration of energy balance, stress resistance, hormone action, and developmental progression.

Trehalose is primarily synthesized in the fat body, an analogous organ to the vertebrate liver and adipose tissue. Trehalose synthesis occurs via a conserved two-step pathway that involves trehalose 6-phosphate synthase (TPS) and trehalose 6-phosphate phosphatase (TPP). TPS catalyzes the formation of trehalose 6-phosphate from glucose 6-phosphate and UDP-glucose, while TPP dephosphorylates trehalose 6-phosphate to produce trehalose (2, 5, 7). Trehalose is then exported into the hemolymph through trehalose transporter 1 (TRET1), to maintain a stable circulating pool that fulfills the demands of the peripheral tissues such as muscles, gut, ovary, etc (8). At the target tissues, trehalose is hydrolyzed by trehalase (Treh), releasing two glucose molecules that enter energy metabolism to support cellular energy demands (5, 7). Yet, this linear description of synthesis, transport, and utilization underestimates the broader regulatory role of trehalose metabolism.

The evolutionary persistence of trehalose lies in its unique physicochemical properties. As a non-reducing disaccharide with an α,α-1,1-glycosidic linkage, trehalose is chemically stable and resistant to glycation and degradation (9). In turn, trehalose is highly resistant to Maillard reaction and non-enzymatic glycation, thus minimizing adverse effects on protein, lipid, and nucleic acid damage that could occur due to interactions between the molecules (9, 10). Notably, it is important for insects because of the presence of the open circulatory system, where tissues are continuously exposed to circulating metabolites. Moreover, the osmotic advantage of trehalose includes the fact that one molecule of trehalose provides an osmotic equivalent of one molecule of glucose, but its hydrolysis provides two glucose molecules, allowing to deliver carbohydrates without the risks associated with osmotic stress (1). Due to these biochemical characteristics and high stability, trehalose is conserved in insects as the major blood sugar.

**Figure 1:**
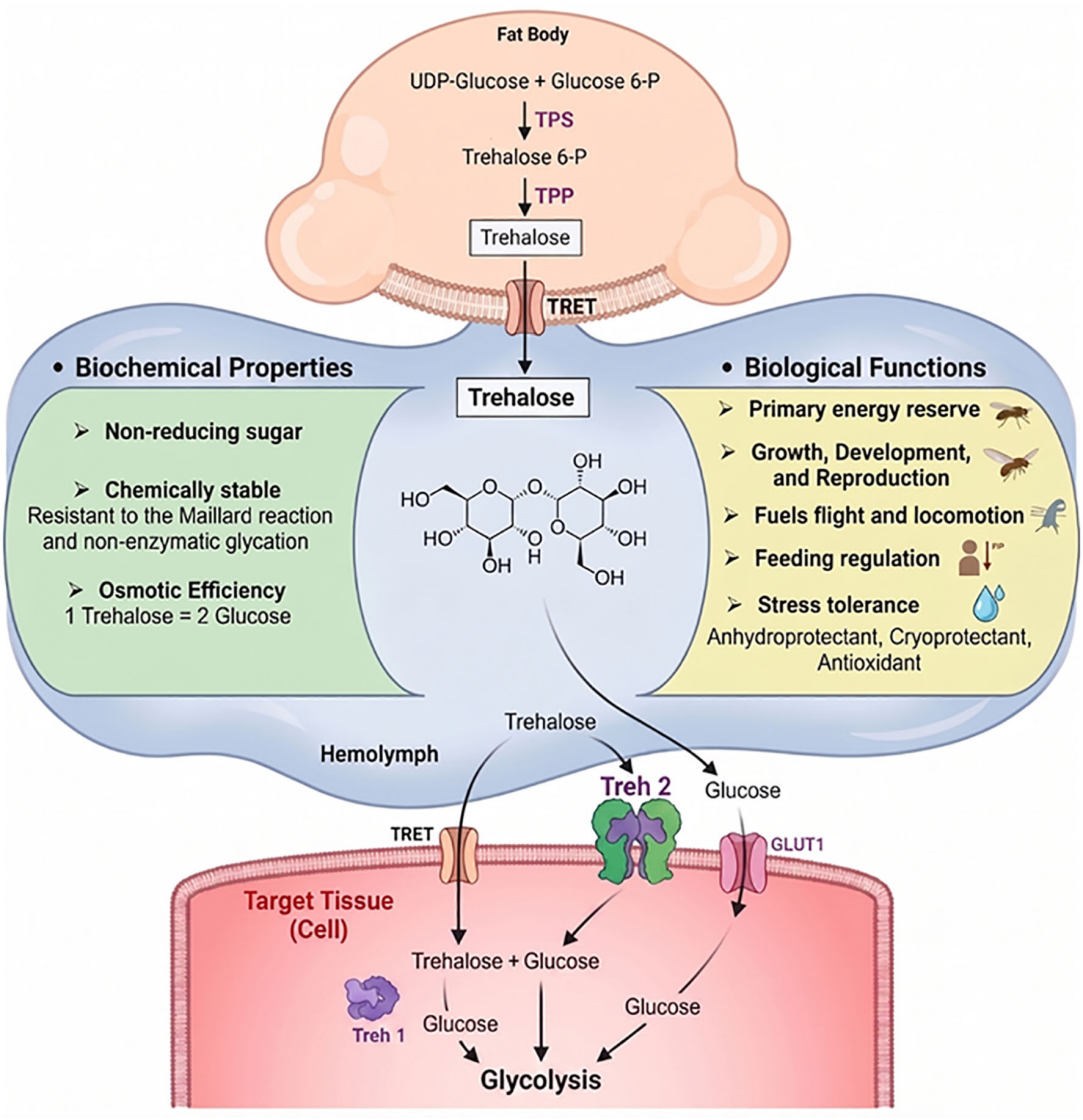
Trehalose metabolic pathway: TPS: Trehalose 6-phosphate synthase, TPP: Trehalose 6-phosphate phosphatase, TREH: Trehalase, TRET: trehalose transporter, GLUT1: glucose transporter 1, HK: Hexokinase. Structure: Schematic representation of trehalose, a disaccharide composed of two α-D-glucose units linked by an α, α-1,1-glycosidic bond (shown in brown box). This unique linkage confers high chemical stability to trehalose. Biochemical properties: non-reducing disaccharide, chemically stable and non-reactive, osmotically beneficial. Multifunctional role in insects: Trehalose functions as a primary energy reserve and a stress-protective molecule, supporting insect development, flight activity, and tolerance to environmental stresses such as cold, desiccation, and oxidative damage.

Through evolutionary adaptation, insects have favored trehalose over glucose for reasons extending beyond metabolic efficiency. Trehalose exhibits unique physicochemical properties that enable insects to withstand extreme environmental conditions. During diapause, desiccation, cold, and thermal stress, trehalose levels increase substantially, serving as a cryoprotectant and anhydroprotectant (11). Trehalose forms a stable, glass-like structure under desiccating conditions, which protects membrane bilayers, prevents protein denaturation, and preserves cellular integrity (11, 12). These properties are particularly important for terrestrial insects exposed to rapid fluctuations in temperature and humidity during development. Consequently, trehalose metabolism supports both energy requirements and cellular defense mechanisms, promoting survival under adverse conditions (**Figure 1**).

Increasingly, trehalose metabolism is being recognized as an important part of the regulatory network that connects energy balance with endocrine signaling and nutrient sensing. Fluctuations in trehalose availability are closely coupled to hormonal pathways, including insulin-like peptides (ILPs), adipokinetic hormone (AKH), juvenile hormone (JH), and ecdysteroids, as well as conserved signaling modules such as Target of Rapamycin (TOR) and AMP-activated protein kinase (AMPK) pathways, as well as the Forkhead box O (FoxO) transcription factors (13). Through these interactions, trehalose metabolism is associated with changes in growth, developmental timing, and survival strategies in response to internal and external cues.

In this review, we synthesize emerging evidence to highlight that trehalose metabolism is a dynamic component of physiological processes that extends beyond its traditional role as an energy reservoir. By framing trehalose as an integrative hub linking metabolism, hormonal regulation, and environmental adaptation, we highlight its central role in insect biology and its potential as a selective target for sustainable pest management strategies.

## 2 Crosstalk of trehalose metabolism with hormones and nutrient-sensing pathway

The metabolic regulation of trehalose plays a key role in systemic energy homeostasis in insects through tight modulation by hormonal and nutritional signals. The AKH stimulates the degradation of glycogen, synthesis, and mobilization of trehalose, leading to increased trehalose levels in hemolymph during starvation and stress (14, 15). On the other hand, ILPs inhibit trehalose synthesis during insect growth and feeding (16). Furthermore, JH and ecdysteroids control trehalose hydrolysis during starvation and thus halt insect development until reach critical body weight (13). All these hormonal activities are regulated by conserved signaling cascades, including TOR, AMPK pathways, as well as the FoxO. Changes in trehalose levels are frequently associated with alterations in TOR, AMPK, and Foxo signaling, which collectively regulate growth, developmental timing, and metabolic adaptations (17–19). Collectively, these observations support a model in which trehalose metabolism is involved in coordinating energy homeostasis with insect development.

### 2.1 Developmental hormones: ecdysteroid and juvenile hormone

Ecdysteroids, particularly ecdysone and its active form 20-hydroxyecdysone (20E), are key steroid hormones that regulate major developmental transitions in insects, including molting and metamorphosis. The timing and magnitude of ecdysteroid pulses are critical for proper progression through developmental stages (20). In *Drosophila melanogaster*, ecdysteroid signaling, in conjunction with the competence factor *fushi tarazu* (*Ftz-F1*), induces the expression of trehalose transporters and Treh enzymes, which promote the breakdown of circulating trehalose. This mechanism generates a transient increase in glucose oxidation, providing the energy required for metamorphosis (21). Importantly, trehalose catabolism has been reported in *D. melanogaster* to influence ecdysteroid biosynthesis and signaling, and facilitate water loss in the puparium, a process essential for successful pupation, suggesting a feedback relationship between carbohydrate metabolism and endocrine signaling (22, 23). Research in *Bombyx mori* and other insects demonstrates that ecdysteroids can suppress carbohydrate hydrolysis during specific developmental stages, such as the penultimate larval instar, thereby modulating monosaccharide concentrations in the hemolymph. The expression of *ecdysone receptor isoforms (EcR)* and *Treh* in tissues such as the silk gland is also temporally regulated by ecdysteroid pulses, underscoring the hormone’s role in coordinating tissue-specific metabolic responses during molting and metamorphosis (24–26).

JH is another developmental cue that functions as a key endocrine regulator by maintaining larval status and preventing premature metamorphosis. JH plays a significant role in controlling trehalose metabolism, particularly during periods of rapid growth and reproductive development (27). In *Tribolium castaneum*, JH modulates trehalose homeostasis through the regulation of Treh enzymes and TRETs. Reduced JH biosynthesis or signaling results in decreased carbohydrate metabolism and increased survival under starvation, highlighting the importance of JH in nutrient utilization and stress resistance (13, 28, 29). In *Helicoverpa armigera*, JH activates *Treh 2* via the GPCR-cAMP-PKA pathway, which enhances trehalose hydrolysis and ensures adequate glucose availability for ovarian development. JH also facilitates Treh 2 binding to TRETs, thereby regulating intracellular sugar concentrations and the transcription of transporter genes (30). The influence of JH on trehalose metabolism extends to developmental timing; disruption of JH signaling alters trehalose turnover, which in turn affects the timing of metamorphosis and stress resilience. The circadian clock further modulates the biosynthesis of JH and ecdysteroids, integrating environmental cues with hormonal and metabolic pathways to optimize developmental processes (29–31). Emerging evidence suggests that changes in trehalose metabolism may influence hormonal synthesis and responsiveness through feedback interactions. Trehalose hydrolysis in *D. melanogaster* facilitates water loss during pupation, indirectly influencing ecdysteroid titers and developmental timing (22). In *Manduca sexta*, trehalose injections can restore imaginal disk growth under starvation conditions, demonstrating that trehalose availability can partially compensate for nutritional limitation under specific conditions (32). These instances suggest that the relationship between trehalose metabolism and hormonal regulation is reciprocal.

### 2.2 Nutrient-sensing and growth pathways

In insects, trehalose acts as an intermediary between carbohydrate status and nutrient sensing through the TOR, AMPK, and insulin/IGF signaling (IIS) cascades (13, 33, 34). The glucose produced from trehalose participates in glycolysis and TCA cycle reactions, altering the cellular glucose/ATP ratio that regulates AMPK activity, an energy sensor responding to cellular energy state and glucose within lysosomes (35, 36). AMPK activation inhibits the action of TOR, inducing catabolism; therefore, changes in trehalose availability are associated with insect developmental and metabolic responses. Moreover, there is a tight connection between IIS and trehalose metabolism. Alterations in ILPs and/or InR activity impact trehalose, glycogen, and fat content and modulate metamorphosis and fecundity, indicating that IIS senses the presence of carbohydrates to integrate growth and reproductive processes (37, 38). For example, *ILP2* knockout affects the trehalose-glycogen-glucose pool and pupation/eclosion success in *T. castaneum* larvae subjected to CO_2_ stress (39). Thus, trehalose availability interacts with and reflects the balance between IIS-TOR (anabolic) and AMPK signaling (catabolic) activities.

**Figure 2:**
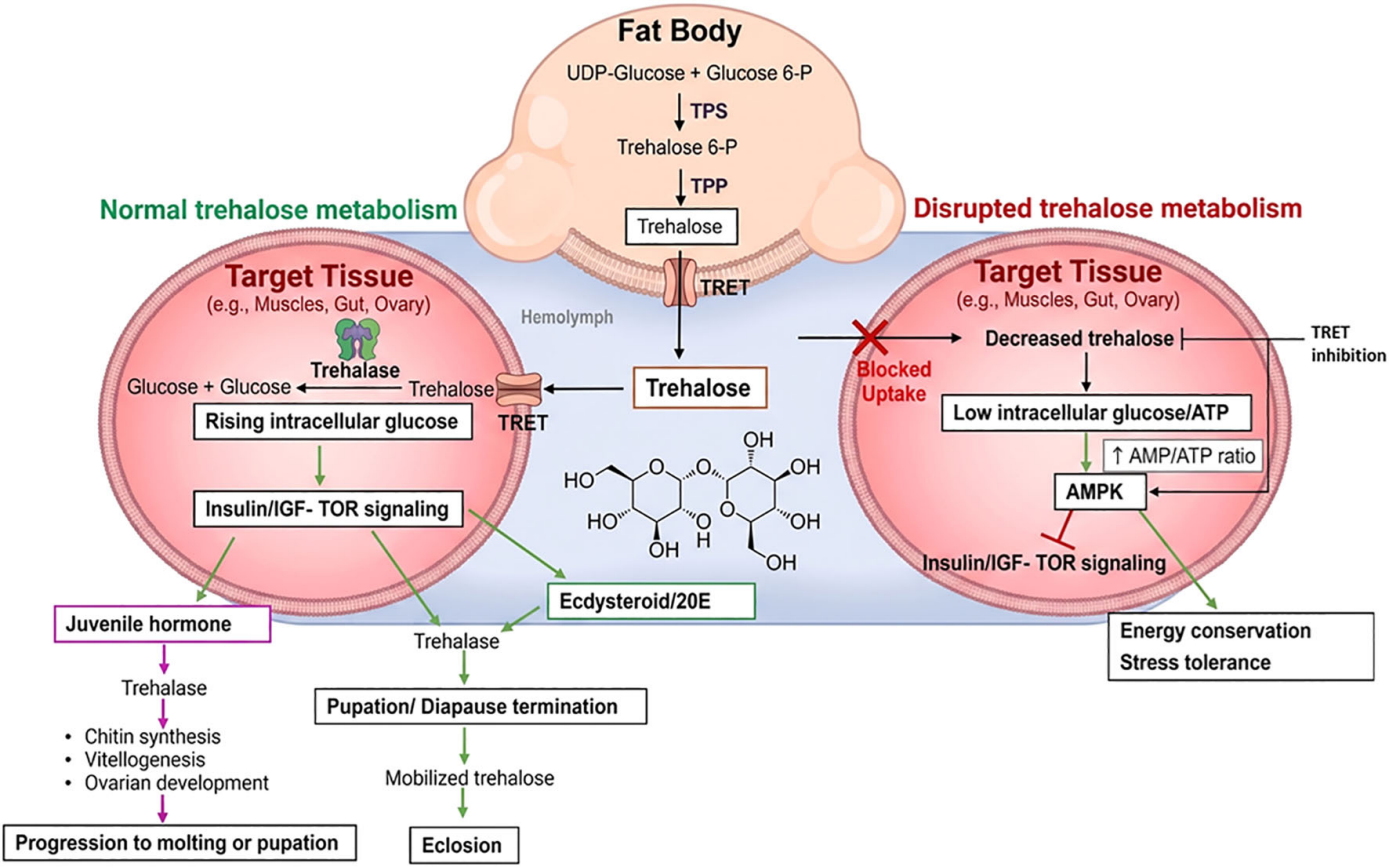
Trehalose metabolism integrates energy homeostasis, hormonal, and developmental regulation in insects. In the fat body, trehalose is synthesized from UDP-glucose and glucose 6-phosphate via trehalose 6-phosphate synthase (TPS) and trehalose 6-phosphate phosphatase (TPP), and released into the hemolymph. Under normal conditions, trehalose is hydrolyzed by trehalase (Treh) in target tissues (e.g., muscle, gut, and ovary), leading to increased intracellular glucose levels and activation of insulin/IGF-TOR signaling. This metabolic state is associated with hormonal pathways, including juvenile hormone and ecdysteroids (20-hydroxyecdysone; 20E), to promote growth, molting, pupation, diapause termination, and eclosion. In contrast, disruption of trehalose metabolism reduces trehalose availability, resulting in low intracellular glucose and ATP levels. This energy deficit activates AMPK signaling while suppressing insulin/IGF-TOR signaling, shifting physiology toward energy conservation and enhanced stress tolerance rather than growth and development. (Both green and purple arrowheads indicates activation and red lines indicate inhibition).

Changes in trehalose metabolism are associated with endocrine and metabolic states that are linked to diapause maintenance, termination, and metamorphic progression. For example, diapause pupae of *Antheraea pernyi have* higher concentrations of trehalose, while diapause termination is marked by increased 20E, induction of *trehalase* (*Treh 1A, Treh 2*) activity, and reduction in trehalose content, pointing towards an active mobilization of trehalose for subsequent development through steroid action (35). Knockdown of *EcR* or blocking its signal via antagonist reduces expression of Treh genes and thereby results in trehalose concentration at a higher level, and prevents eclosion, suggesting that EcR-dependent trehalose catabolism contributes to the metabolic conditions associated with diapause termination (35). IIS components provide another layer of control over metabolism. In *A. pernyi*, during diapause, trehalose is stored as a protected resource under reduced IIS and altered JH/ecdysteroid titers. The administration of insulin or induction of endogenous ILPs promotes activation of trehalases and trehalose degradation, facilitating adult eclosion, while *ILP* knockdown or overexpression of Foxo maintains trehalose levels and delays emergence (40). These studies indicate that IIS regulates the trehalose catabolic rate, thereby modulating the metabolic state at which ecdysteroid pulses can successfully promote metamorphosis. In *Bactrocera minax*, disruption of the 20E biosynthesis gene *CYP314A1* alters trehalose, glycogen, and lipid levels, leading to changes in diapause and post-diapause development. This finding further supports the connection between energy balance and ecdysteroid thresholds (41). Altogether, high-trehalose, low-catabolism states promote diapause maintenance, while endocrine signals that stimulate trehalose breakdown facilitate developmental progression toward molting and pupation. Crosstalk of trehalose metabolism with hormones and nutrient-sensing pathways discussed above are summerized in **Figure 2**.

## 3 Developmental consequences of perturbed trehalose metabolism

Trehalose metabolic genes, *TPS/TPP* and *Treh*, play a critical role in regulating larval growth, molting, and metamorphosis in insects. Disruption of this metabolic pathway demonstrates that metabolic homeostasis is fundamental to successful developmental transitions. Multiple studies demonstrate that both genetic and pharmacological perturbations alter the expression and activity of trehalose metabolic enzymes. This alteration disrupts glucose availability, glycogen metabolism, and chitin pathway gene expression, thus causing abnormal molting, metamorphosis, and high mortality rates. **Table 1** summarizes representative studies across insect species, highlighting conserved and divergent outcomes of trehalose metabolic dysregulation.

**Table 1:** Developmental consequences of perturbed trehalose metabolism.

Insect species	Target gene and type of perturbation	Metabolic effect	Developmental/physiological outcome	Key insight	Reference
*Leptinotarsa decemlineata*	TPS RNA interference (RNAi)	↓ Trehalose	Larval/pupal lethality	Trehalose synthesis is indispensable for survival	(42)
*Leptinotarsa decemlineata*	TPS RNAi Survivors	↑ Glycogen, ↑ Lipids, ↑ Proline, ↓ Chitin	Increased feeding, growth defects; rescued by trehalose supplementation	Trehalose deficiency triggers compensatory metabolic reprogramming	(42)
*Heortia vitessoides* *Hyphantria cunea*	TPS RNAi	↓ Trehalose; ↓ Chitin pathway genes	Pupal deformities, wing defects, incomplete ecdysis	Trehalose metabolism regulates chitin biosynthesis genes	(43, 44)
*Drosophila melanogaster*	Tps1 Loss-of-function mutant	Reduced hemolymph trehalose	Growth defects, pre-eclosion lethality, loss of homeostasis	Trehalose is critical for systemic metabolic balance	(36)
*Helicoverpa armigera*	TPS/TPP RNAi	↓ Trehalose metabolism, ↓ Glycolysis	Defective eclosion, abnormal muscle structure	Trehalose regulates E2F–Mef2 axis controlling muscle development	(45)
*Triboleum castaneum*	TPS RNAi	↓ Trehalose, ↓ Chitin	Failure to molt (larva → pupa), abnormal molting phenotypes, high mortality	TPS regulates chitin biosynthesis and is essential for successful molting	(46)
*Nilaparvata lugens*	TPS RNAi	↓ Glycogen; altered glycogen metabolism	Abnormal phenotypes, increased moulting and wing deformities, and mortality rates	Trehalose-glycogen pools are reciprocally regulated	(47)
*Acyrthosiphon pisum Harris*	TPS/Treh RNAi	↓ Chitin; altered expression of chitin metabolism genes	Molting deformities (both morphs), wing deformities (red morph), and abnormal development	TPS and TREH regulate chitin metabolism at the transcriptional level and are essential for normal metamorphosis	(48)
*Helicoverpa armigera, Spodoptera frugiperda*	TPP Inhibitor treatment: N-(phenylthio)phthalimide	↓ Trehalose metabolism, ↓ Glycolysis, ↓ TCA intermediates	Reduced larval size, failure to pupate	Energy collapse underlies developmental arrest	(49)
*Mythimna separata*	Treh (soluble + membrane-bound) RNAi	↑ Trehalose, ↑ Glycogen, ↓ Glucose, ↓ Treh activity, ↓ Chitin	Severe molting defects, thin cuticle (stronger with Treh1 knockdown)	Trehalose hydrolysis is essential for chitin synthesis and molting	(50)
*Leptinotarsa decemlineata*	Treh 1a/Treh 2 RNAi	↑ Trehalose	Lethality	Excess trehalose is also toxic → tight regulation required	(42)
*Triboleum castaneum*	Treh RNAi	↑ Trehalose, ↓ Glucose, ↓Glycogen, ↓ Chitin	Molting defects, cuticle abnormalities, lethality	Impaired trehalose hydrolysis disrupts chitin biosynthesis (via glucose supply)	(33)
*Helicoverpa armigera*	Treh Inhibitor treatment: Validamycin A	↓ Glucose, ↓ Glycolytic & TCA intermediates, Activation of alternative metabolism pathways	Systemic energy deficiency: Shift to protein/lipid catabolism	Energy stress triggers metabolic rewiring	(51)
*Nilaparvata lugens, Spodoptera litura*	Treh Inhibitor treatment: Validamycin A	↑ Trehalose, ↓ Glucose, ↓ Chitin	Defective wing formation, incomplete ecdysis	Trehalose breakdown linked to cuticle formation and wing morphogenesis	(52, 53)
*Aedes aegypti*	Treh Inhibitor treatment: Validamycin A	↑ Trehalose imbalance	Delayed development, reduced flight, shorter lifespan	Trehalose is essential for flight muscle energetics	(54, 55)
*Drosophila melanogaster*	Treh mutants-Trehalose feeding	↑ Trehalose toxicity	Poor diet adaptation, reduced viability	Both excess and deficiency are harmful	(56)

Evidence from multiple insect species about genetic and pharmacological perturbations of trehalose metabolism shows that both depletion and accumulation of trehalose disrupt metabolic balance and developmental processes.

Collectively, numerous studies on various insects have shown that normal regulation of trehalose metabolism is essential for proper growth, moulting, metamorphosis, and survival. Disturbance in trehalose synthesis or degradation usually leads to developmental defects, including incomplete molting, defective ecdysis, wing defects, impaired eclosion, and increased mortality. These phenotypes are commonly associated with the changes in glucose levels, glycogenesis, and chitin production, thus emphasizing the significance of trehalose homeostasis and its relation to development. Notably, impairment in both trehalose synthesis and hydrolysis can be detrimental, indicating that normal development depends on maintaining trehalose levels within an appropriate physiological range. Moreover, recent metabolomics analysis indicates that disturbance in trehalose metabolism triggers alterations in carbohydrate, lipids, and protein metabolism, showing disturbances in energy homeostasis (42). Altogether, these studies highlight the significance of trehalose metabolism in providing energy needs for normal development.

## 4 Emerging perspectives and open questions

Future research on trehalose metabolism in insects should focus on the regulatory role of trehalose 6-phosphate as a developmental signal. Although this has been extensively studied in plants, it remains poorly explored in insects. Advanced spatiotemporal approaches, such as omics and imaging, will clarify its tissue- and stage-specific dynamics. The trehalose pathway is a promising target for pest control. This is due to its essential role in insects and its absence in vertebrates. RNAi and inhibitors show potential for control, though specificity and delivery are still challenging. However, despite all these advancements, there are several hurdles that have to be overcome before the metabolism of trehalose can be successfully used for controlling pests in the field. First of all, RNAi technology often shows species-specific variability in efficiency, which is associated with differences in double-stranded RNA (dsRNA) absorption, stability, and systemic transport among insects. In addition, prolonged treatment with trehalose metabolic inhibitors can induce selection pressures that, in turn, lead to the development of resistance, either through modification of the target site or through alternative metabolism. Since the mechanism of trehalose metabolism is highly conserved among different insects, the possible effects on non-target species, such as pollinators, predators, and parasitoids, should be carefully assessed as well. Environmental and nutritional factors also significantly influence trehalose metabolism. This highlights the importance of field-based and ecological studies. Integrative approaches will support effective and targeted pest management strategies.

An important unresolved question is whether developmental defects resulting from perturbation of trehalose metabolism arise from trehalose-specific signaling functions or from broader energetic and nutritional imbalances. Many studies demonstrate strong associations between altered trehalose homeostasis, endocrine signaling, and developmental outcomes; however, direct causal evidence establishing trehalose as a direct signaling molecule remains limited. Future studies employing tissue-specific genetic manipulation, metabolite tracing, and temporally resolved analyses will be required to distinguish putative signaling functions from secondary consequences of metabolic stress.
